# Supplementary material for: Adenosine and Cordycepin Accelerate Tissue Remodeling Process through Adenosine Receptor Mediated Wnt/β-Catenin Pathway Stimulation by Regulating GSK3b Activity
Source: Int J Mol Sci. 2021 May 25;22(11):5571. doi: 10.3390/ijms22115571 (PMC8197479; doi:10.3390/ijms22115571)
Supplement: Supplementary file 1 [file ijms-22-05571-s001.zip › Supplementary Information_Cordyceps militaris Extract.pdf]

Supplementary Information

1. Constituents analysis for Cordyceps militaris extract

To identify constituents of *Cordyceps militaris*(CM) extract, HPLC analysis was performed. The dried CM were extracted with distilled water (1:15) for 1 hour at 121℃, filtered through Whatman No. 4 filter paper. The filtrate was concentrated by rotary evaporator under reduced pressure.. The extract was reconstituted in water. For quantitation, an external standard method was utilized. Adenosine, cordycepin, guanosine and uridine were used as standards of CM extract as previously reported. The stock solutions of each standard were prepared in water just before use. The standards were purchased from Sigma (St. Louis, MO, USA, adenosine, cordycepin, and guanosine) and TCI (Tokyo, Japan, uridine). Following the previous reports [1], the analytical conditions were described in Table S1. Figure S1 shows the HPLC profiles of the standards (a-d) and CM Extract (e) at 260 nm. The contents and retention times of the compounds were showed in Table S2. As the result of the analysis, adenosine (177 μg/mL), guanosine (85 μg/mL), uridine (65 μg/mL), and cordycepin (7.9 μg/mL) were detected in the extract of *Cordyceps militaris*.

Table S1. Analytical conditions of HPLC analysis

<table>
<tr><th>HPLC test method</th><th>Time
(min)</th><th>Flow rate
(ml/min)</th><th>Water (%)</th><th>MeOH
(%)</th></tr>
<tr><td rowspan="6">- Agilent 1260 Infinity system

- Column: Agilent Eclipse XDB-C18
(4.6 × 250 mm, 5 μm)

- Column temperature: 25℃

- Injection volume: 1 μl

- Detector: HPLC-DAD (260 nm)

- Eluent</td><td>0</td><td>1.0</td><td>95</td><td>5</td></tr>
<tr><td>30</td><td>1.0</td><td>40</td><td>60</td></tr>
<tr><td>31</td><td>1.0</td><td>0</td><td>100</td></tr>
<tr><td>41</td><td>1.0</td><td>0</td><td>100</td></tr>
<tr><td>42</td><td>1.0</td><td>95</td><td>5</td></tr>
<tr><td>57</td><td>1.0</td><td>95</td><td>5</td></tr>
</table>

Table S2. Contents of Compounds in the *Cordyceps Militaris* Extract

No.	Compound	Content (μg/mL)	t_R (min)
1	Uridine	65	6.0
2	Guanosine	85	8.1
3	Adenosine	177	11.9
4	Cordycepin	7.9	13.0

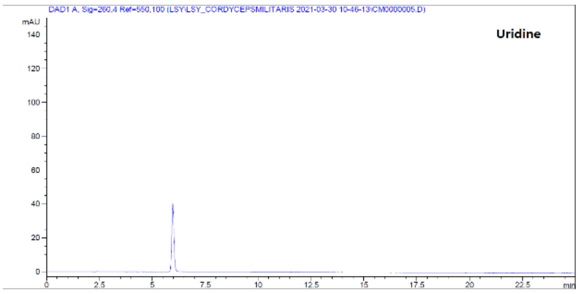

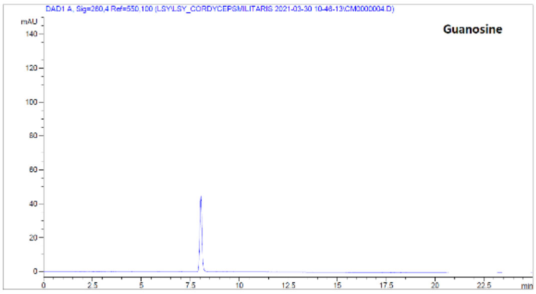

(a) **(b)**

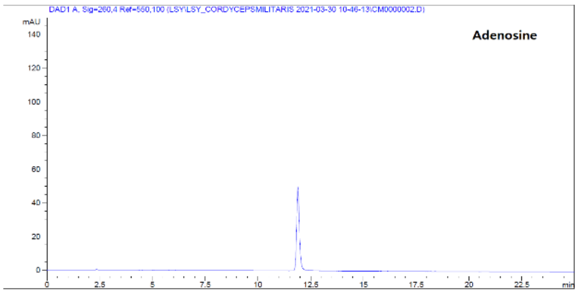

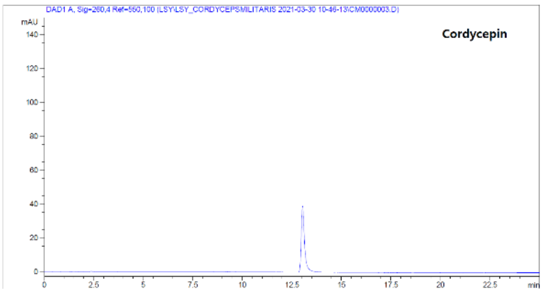

(c) **(d)**

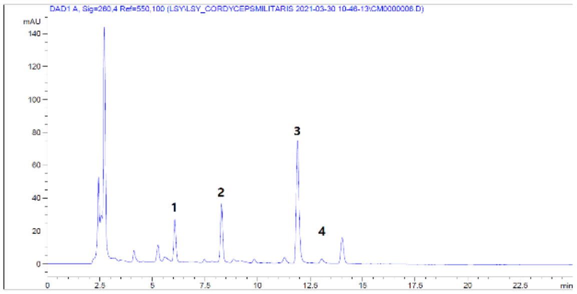

(e)

Figure 1S. HPLC profiles of the standards (a-d) and compounds in *Cordyceps Miliatris* Extract (e) at 260 nm, respectively. The CM extract was diluted 5% in water. Peaks: (1) uridine, (2) guanosine, (3) adenosine, (4) cordycepin.

References

1. Yu, R.; Ye, B.; Yan, C.; Song, L.; Zhang, Z.; Yang, W.; Zhao, Y., Fingerprint analysis of fruiting bodies of cultured Cordyceps militaris by high-performance liquid chromatography–photodiode array detection. *Journal of pharmaceutical and biomedical analysis* **2007,** 44, (3), 818-823.
